# A Case of Retention of Urine, in Which a Puncture of the Bladder, in the Hypogastric Region, Proved, under Very Unpromising Circumstances, Successful; to Which Are Added Three Cases of Retention of Urine, and Some Remarks on Diseases of the Urinary Bladder

**Published:** 1790

**Authors:** James Lucas

**Affiliations:** one of the Surgeons to the General Infirmary at Leeds


### LONDON MEDICAL JOURNAL.

### I. *A Case of Retention of Urine, in which a Puncture of the Bladder, in the hypogastric Region, proved, under very unpromising Circumstances, successful; to which are added three other Cases of Retention of Urine, and some Remarks on Diseases of the urinary Bladder. By Mr.* James Lucas, *one of the Surgeons to the General Infirmary at Leeds.*

A Stout man, of the first battalion of West-Riding militia, aged thirty years, was admitted into our Infirmary on the 1st of June, 1775, with a painful abscess *in perinæo*, and a circumscribed intumescence extending from the pubis to the umbilicus.

His tongue was white, and foul, though he had not much heat, nor did his pulse exceed ninety strokes in a minute: he was costive, and could not void any urine; had frequent hiccough, and a constant difficulty of breathing, accompanied with sickness and languor: he complained of an itching in the skin, and of excessive pain throughout the urinary passages.

For

For the particulars of the cafe, previoufly to the admiffion of the patient into the Infirmary, I was indebted to Mr. Walker, the regimental furgeon, who informed me, that, on the 17th of May, the patient, in leaping over two chairs, placed with their backs together, had fallen acrofs them, and bruifed the perinæum in fo violent a manner as to produce an immediate fainting; that a few hours after, being under the neceffity of attending the parade, he had been feized, whilft in the ranks, with a profufe hæmorrhage from the urethra, fucceeded by acute pain, and in twenty-four hours by a total retention of urine; that at this period of the cafe the late Mr. Billam, formerly fenior furgeon of the Infirmary at Leeds, had been confulted; that every attempt to pafs a catheter or bougie had failed; but that by a ftrict antiphlogiftic treatment the fymptoms had gradually abated, and in four days the man feemed to be perfectly recovered.

Mr. Walker added, that on the 28th of May the pain and fwelling of the perinæum and abdomen had returned, and the urine paffed involuntarily; that on the 31ft it ceafed to be in any way evacuated, and that the patient now appeared appeared to receive no benefit from thofe remedies which had before proved fo effectual.

Mr. Walker attributed this relapfe to intemperance.

A confultation of the phyficians and furgeons of the Infirmary was called, and the gentlemen, who had previoufly attended the patient, were requefted to continue their vifits. To all of them I was much indebted for their judicious affiftance; nor fhould I omit to mention the evident advantage that arofe in this cafe from the difcernment and affiduity of Mr. Carr, the houfe apothecary.

A careful examination was made by placing the patient in different poftures, but particularly in the pofition for lithotomy. The abfcefs *in perinæo* was opened on the day of his admiffion, and a quantity of pus was difcharged. Ineffectual attempts were made to pafs fmall catgut through the wound into the urethra; it not having been deemed advifable to extend the incifion towards the bladder. The introduction of bougies and catheters along the natural paffage, during the ufe of the warm bath, proved alfo inefficacious; as did likewife the ufe of topical fomentations, of purgative and opiate clyfters, and of mercurial, cathartic, and and anodyne medicines, all of which had a fair trial.

Under thefe circumftances, all the gentlemen who favoured me with their attendance concurred with me in propofing the puncture of the bladder in the region of the pubis; but to this the patient obftinately refufed his affent, during the fpace of nearly forty-eight hours, although he continued in great pain.

A little urine fometimes involuntarily efcaped through the urethra, but never in fufficient quantity to mitigate his pain, or to diminifh the fize of the abdomen, the fwelling of which was now extended nearly to the *fcrobiculus cordis*.

At length, on the 3d of June, when the difeafe had made fo great a progrefs that his death was hourly expected, the patient expreffed a wifh, that if any chance of fuccefs remained, the operation might be performed.

I accordingly punctured the bladder, in the middle of the hypogaftric region, about two inches above the fymphyfis of the pubis, and fix pints of high-coloured urine were difcharged. Two inches of the canula (which was not fo convenient a one as might have been contrived for the purpofe) were retained in the wound, and the urine was prevented from being evacuated, cuated, except at ſuch times as might be deemed neceſſary, by ſtopping the mouth of the inſtrument with a cork. A piece of ſponge was applied around the canula, to hinder excoriation from any urine that might eſcape at the ſides of the inſtrument. The whole of the dreſſings were ſecured by a T bandage with ſcapulary ſtraps.

Although conſiderable eaſe had been obtained by the operation, yet the dangerous ſtate to which the patient had been previouſly reduced rendered the ſubſequent treatment an object of equal attention.

As near four pints more of urine were again taken off the ſame evening, it appeared probable that the bladder had not before been equally emptied. A moſt refreſhing ſleep commonly ſucceeded each evacuation of urine.

June 6th, The canula was found to have eſcaped during the night; and having failed in my endeavours to replace it, I introduced a ſhort female catheter, which I retained as firmly as I could.

—— 7th, The patient began to complain of ſlight but painful efforts to void urine in a natural way, by which we were encouraged to attempt the paſſage of bougies and catgut by the urethra, as well as by the wound *in perinæo*;

 but but the pain brought on by thefe trials prevented our perfifting.

June 20th, The urine began to force its way in fmall drops through the wound *in perinæo,* although this was fo nearly healed that the fmalleft catgut could not be introduced into it.

—— 26th, A very fmall bougie was paffed along the urethra until it occafioned a flow of urine; but fuch intolerable and permanent pain enfued, that after this we relied upon retaining the urine fo long in the bladder as to invite, if not compel, it to pafs through the more depending channel, until its difcharge by the catheter became lefs and lefs requifite.

July 20th, The bladder being entirely emptied by the depending paffages, and chiefly by the urethra, the catheter was withdrawn.

The patient gradually regained his ftrength, the wounds perfectly healed, and on the 18th of Auguft he was difcharged from the Infirmary cured.

In this cafe the idea of puncturing the bladder through the rectum did not occur to any of us. From the frequent and ftrict examinations of the patient, and the free paffage of the clyfters, I am inclined to think there was not any confiderable fwelling in that gut, efpecially as

I have

I have ſince, in caſes where the bladder was much diſtended, been unable to find ſo much fulneſs as I ſhould have expected.

The power of gradually forcing the urine to take its natural courſe ſeems to be a material advantage attending the puncture employed in the preſent caſe; and perhaps a variety in the caſe may render each operation to become with equal propriety preferred.

The ſmooth catheters now in uſe, as well as canulas made of the ſame materials, might often be advantageouſly employed in preventing the painful ſenſations which are frequently occaſioned by a metallic ſubſtance touching tender membranous parts.

I have frequently obſerved in other inſtances, as well as in the preſent caſe, how ill thoſe who have been ſubject to complaints in the urinary paſſages bear any exceſs.

The patient returned to the Infirmary about a year after with a fiſtula *in perinæo*, and was in a fair way of recovery, had not his habits of intemperance obliged the truſtees to diſmiſs him.

### CASE II.

June the 1ſt, 1770, the coachman of a gentleman in this town conſulted an empiric for the cure of a recent gonorrhœa. From the account given by the patient, not only draſtic cathartics, but ſtimulating diuretics, had been repeatedly adminiſtered, until an acute pain in the urethra, a violent fever, and a total retention of urine, had ſo far alarmed the quack, that he begged the future management of the caſe might be ſubmitted to a regular ſurgeon; and accordingly, on the 23d, Mr. Billam, ſenior ſurgeon of our Infirmary, was conſulted. A cooling regimen, bleeding, a warm bath, aperients, opiates, and other remedies were directed, with a view to remove violent internal inflammation; and the following morning near ſix pints of urine were drawn off by the catheter.

June 25th, The unremitting urgency of the ſymptoms, together with the difficulty of introducing the catheter, induced Mr. Billam to requeſt that I would meet him in conſultation. It was agreed that the perinæum ſhould be fomented, and that a clyſter, containing ſix grains of opium, ſhould be injected. As ſoon as the appeaſing effect of the anodyne remedy was obvious, an attempt was made by Mr. Billam to paſs the catheter, which he obſerved was introduced with much more facility than on the preceding preceding day. At firſt there flowed a well-digeſted pus, and afterwards urine mixed with a bloody matter.

When the diſcharge had ceaſed, the bladder did not appear to be emptied, as an evident fulneſs in the abdomen remained.

By perſiſting in the ſame plan the ſymptoms gradually ſubſided, and on the 28th the patient voided a quart of urine; and from that time his recovery met with no interruption.

From an inquiry ſome time after, it appeared that no obſtruction in the urethra had formed in conſequence of the violent inflammation. The ſuppuration which took place ſeemed to depend more upon the means preſcribed than upon the original diſorder.

Some merit was certainly due to the empiric for reſigning his patient, when we conſider the miſchievous conſequences that have happened from the uſe of the catheter in the hands of ignorant practitioners. An haſty introduction of ſuch an inſtrument, previouſly to the uſe of anodyne and relaxing remedies, might not only have failed, but have proved highly prejudicial.

When a paſſage for the urine cannot, in ſimilar caſes, be obtained, a puncture of the bladder ſhould

ſhould be recommended; and in the choice of the methods adviſed, the cauſe of the obſtruction ſhould be duly attended to. Such affections may ariſe from a morbid ſtate of the proſtate gland, or from an excreſcence at the neck of the bladder, or within its cavity, ſo as to prevent any advantage from the catheter.

### CASE III.

A boy, aged four years, was brought, upon my day of admiſſion, to our Infirmary with a violent inflammation extending over the penis, ſcrotum, and hypogaſtric region, in all of which was a conſiderable intumeſcence. A mortification had taken place in the penis, and many of the inflamed parts were in a ſloughy ſtate. He had an acute fever, complained of exceſſive pain, and had, for three days, diſcharged no urine.

The account given by his parents was, that four days before he had begun to complain of an uneaſineſs in his belly, the ſkin of which had become red, and that the redneſs had gradually ſpread over his private parts.

I paſſed a probe into the urethra, and ſoon diſcovered the original cauſe of his diſorder to be a ſtone in the paſſage. I made an inciſion upon the the ſtone, and it was immediately forced out of the wound by a guſh of urine that continued to flow until the bladder was emptied. The change of countenance in the child was truly expreſſive of the eaſe which he had obtained; and though he ſuffered much afterwards, yet he ſeemed to retain a regard for me during his cure. The ſtone was of a firm texture, and of nearly the ſize of a nutmeg.

One half of the penis, the integuments over the teſticles, and for ſome diſtance over the pubes, were totally deſtroyed, and ſeparated; ſo that a moſt painful ulcer, of large extent, rendered his cure tedious.

In the courſe of the treatment the advantages that were experienced from the fermenting cataplaſm, as well as from liberal and repeated doſes of the tincture of opium, deſerve to be remarked.

It did not appear that the boy had previouſly ſuffered much from the ſtone in his bladder; but the conduct of the parents might make their account leſs to be depended upon, for they had been ſo careleſs as to permit their child to remain four days in extreme pain, although they reſided in Leeds, and might at any hour hour have had medical affiftance without expence.

The great refolution fhewn by this little patient, during his refidence in the hofpital, might not only be a proof of his averfion to complain, but of his having been accuftomed to bear pain.

The erroneous narrative given by the parents of the child may be mentioned as a caution to young practitioners not to depend too much upon fuch accounts, left a failure in inveftigating the real caufe fhould miflead them in their practice, and prove fatal to the patient.

An operation upon a part, in fuch a morbid ftate, might frequently be deemed exceptionable, although in the prefent inftance advifable; as, in all probability, the death of the patient muft have enfued before the caufe of the malady could have been otherwife removed.

The ftone muft in this cafe have rapidly paffed fo far into the urethra as entirely to obftruct the difcharge of urine; for ftones will often remain there as long a time, and will pafs through the urethra, or fo far diftend the paffage, as to permit urine to be evacuated.

In May, 1773, the fon of J. Richardfon, of Holbeek, was admitted into the Infirmary for a

 tumour tumour upon the penis. He was about five years of age, and, from his ſymptoms, it appeared that the ſwelling contained a ſtone, which had by ſlow degrees paſſed from the bladder, and either diſtended or eſcaped out of the urethra. An inciſion, of the length of the tumour, ſoon made it appear that the conjecture was well founded, as a ſtone of the ſize of a pigeon's egg was diſcharged; and the urine flowing out of the wound made it certain that its ſituation was connected with the urethra. By the aid of adheſive plaſters the edges of the wound united in a few days. The tumour was ſaid by the patient's friends to have been obſerved two years before, and appeared to them to have increaſed in ſize.

### CASE IV.

The ſingular affection of the bladder, together with the ſpeedy method of cure, have appeared to me circumſtances ſo far varying from thoſe valuable hiſtories communicated by Dr. Gilchriſt *, as to render a narrative of the ſubſequent caſe not unworthy of notice:

Mr. C., a farmer, of about fifty years of

* Eſſays Phyſ. and Liter: Vol. III. page 471.

age, was, on the 1ſt of Auguſt, 1789, ſeized with a violent pain in his right leg, a complaint to which he had before been ſubject. The pain gradually aſcended to his back, regularly increaſing in the night, and abating in the day time. Some temporary relief had been obtained by bleeding, ſudorifics, and the warm bath; but on the 18th, in the night, the pain extended acroſs the hypogaſtric region, and was attended with frequent ineffectual efforts to paſs urine. On the 19th, although he had a total retention of urine, accompanied with a conſiderable intumeſcence in the regio pubis, yet the diſtance he was at from Leeds, and the hope of becoming eaſier, prevented his applying for farther aſſiſtance until the 20th; when, in addition to his former complaints, he had great heat and thirſt. Upwards of two quarts of urine were then immediately drawn off by means of a catheter made of elaſtic gum. When the urine was evacuated, and the ſwelling of the abdomen had ſubſided, a ſoreneſs and thickening of the bladder might be clearly diſcovered. Due attention was paid to the uſe of aperient and anodyne remedies; and a bolus, compoſed of ten grains of calomel and one grain of opium, was given at bed time.

Although

Although he paſſed a much eaſier night, yet no urine having been evacuated, the catheter was again had recourſe to on the 21ſt, and the bolus of calomel and opium was directed to be repeated both morning and evening.

On the 22d he was not only free from any complaint in the urinary paſſages, but the pain in his limb and back was greatly abated, and never returned with any degree of violence.

From a ſtrict attention to the treatment, I have no doubt but the liberal uſe of mercury, combined with opium, had a principal ſhare of merit in not only removing the inflammatory affection of the bladder, but alſo in alleviating the pain that preceded it*.

The catheter made of gum tangio is, in general, preferable to one made of ſilver; and though no inconvenience commonly enſues from its remaining a few days in the bladder, yet patients, in general, ſeem better ſatisfied with its being occaſionally introduced.

A neceſſity for repeating the uſe of the catheter is often pointed out by efforts to paſs

* The uſe of Buxton waters, which was recommended to this patient in the autumn, ſeemed ſerviceable to his general health; and he has had no return of his diſorder.

urine, or by an uneafinefs in the region of the bladder; and care fhould be taken that no confiderable diftention of it may take place.

In the preceding cafe, the facility with which the inftrument was introduced fhewed that the paffage to the bladder had not become affected, but was free from inflammation — a diftinction in many cafes highly requifite.

I have feen feveral cafes fimilar to thofe related by Dr. Gilchrift. The fubjects of them have generally been males of fixty years of age or upwards, and the complaint has advanced gradually, and required frequent extraction of the urine.

In fuch cafes great dependence may be had upon a liberal exhibition of mercury, though fmall dofes would be of little fervice; and the medicine fhould not be long together omitted until the diforder is in a great meafure removed.

I have known this remedy efficacious in a patient of feventy years old, and in another who had three attacks of a complaint of this kind at fome diftance of time from each other. In very old perfons, apparently worn out in conftitution, failure may reafonably be expected.

Whoever confiders the ftructure of the parts will readily conceive the irreparable injury that may be done in ſuch caſes by illiterate practitioners.

*Leeds,*
February 19, 1790.

